# Effect of image compression and scaling on automated scoring of immunohistochemical stainings and segmentation of tumor epithelium

**DOI:** 10.1186/1746-1596-7-29

**Published:** 2012-03-21

**Authors:** Juho Konsti, Mikael Lundin, Nina Linder, Caj Haglund, Carl Blomqvist, Heli Nevanlinna, Kirsimari Aaltonen, Stig Nordling, Johan Lundin

**Affiliations:** 1 Institute for Molecular Medicine Finland (FIMM), P.O. Box 20, FI-00014 University of Helsinki, Helsinki, Finland; 2 Department of General Surgery, Helsinki University Central Hospital, Helsinki, Finland; 3 Department of Oncology, Helsinki University Central Hospital, Helsinki, Finland; 4 Department of Obstetrics and Gynaecology, Helsinki University Central Hospital, Helsinki, Finland; 5 Department of Pathology, University of Helsinki, Helsinki, Finland; 6 Department of Clinical Genetics, Helsinki University Central Hospital, Helsinki, Finland

**Keywords:** Breast cancer, Colorectal cancer, Immunohistochemistry, Texture analysis, Image processing, Computer-Assisted, Image compression, Image scaling

## Abstract

**Background:**

Digital whole-slide scanning of tissue specimens produces large images demanding increasing storing capacity. To reduce the need of extensive data storage systems image files can be compressed and scaled down. The aim of this article is to study the effect of different levels of image compression and scaling on automated image analysis of immunohistochemical (IHC) stainings and automated tumor segmentation.

**Methods:**

Two tissue microarray (TMA) slides containing 800 samples of breast cancer tissue immunostained against Ki-67 protein and two TMA slides containing 144 samples of colorectal cancer immunostained against EGFR were digitized with a whole-slide scanner. The TMA images were JPEG2000 wavelet compressed with four compression ratios: lossless, and 1:12, 1:25 and 1:50 lossy compression. Each of the compressed breast cancer images was furthermore scaled down either to 1:1, 1:2, 1:4, 1:8, 1:16, 1:32, 1:64 or 1:128. Breast cancer images were analyzed using an algorithm that quantitates the extent of staining in Ki-67 immunostained images, and EGFR immunostained colorectal cancer images were analyzed with an automated tumor segmentation algorithm. The automated tools were validated by comparing the results from losslessly compressed and non-scaled images with results from conventional visual assessments. Percentage agreement and kappa statistics were calculated between results from compressed and scaled images and results from lossless and non-scaled images.

**Results:**

Both of the studied image analysis methods showed good agreement between visual and automated results. In the automated IHC quantification, an agreement of over 98% and a kappa value of over 0.96 was observed between losslessly compressed and non-scaled images and combined compression ratios up to 1:50 and scaling down to 1:8. In automated tumor segmentation, an agreement of over 97% and a kappa value of over 0.93 was observed between losslessly compressed images and compression ratios up to 1:25.

**Conclusions:**

The results of this study suggest that images stored for assessment of the extent of immunohistochemical staining can be compressed and scaled significantly, and images of tumors to be segmented can be compressed without compromising computer-assisted analysis results using studied methods.

**Virtual slides:**

The virtual slide(s) for this article can be found here: http://www.diagnosticpathology.diagnomx.eu/vs/2442925476534995

## Background

Computer-assisted quantification of biomarkers in biological tissue samples is becoming increasingly utilized [1-5], due to objectivity, repeatability and savings in human labor. Meanwhile, the use of whole slide scanning of pathological tissue glass slides is producing large image files up to hundreds of gigabytes in uncompressed format, which have to be stored in digital slide archives. The size of these archives including back-ups requires extensive storage capacity. To reduce the need for extensive data storage systems image files can be compressed and scaled down.

Not only storage space is affected by image compression and scaling, but also the bandwidth needed for transfer of images is diminished. In contrast, compression and scaling requires more processing power initially, as do the decompression of images when viewed or analyzed. Scaling speeds up automated quantification and classification algorithms.

Generally, in order not to lose image data, the recommendation for optimal image analysis has been to use uncompressed images without image scaling [6]. However, studies have shown that image compression has only minor effect on visually performed diagnostics [7] and computer assisted image analysis [8]. The role of compression in automated quantification of immunohistochemical (IHC) stainings has been assessed in a few previous publications [9-11]. Image scaling and visual image quality has been addressed in the literature [12], but to our knowledge, none of the previous studies has taken the effect of image scaling on automated image analysis into account.

**Figure 1:**
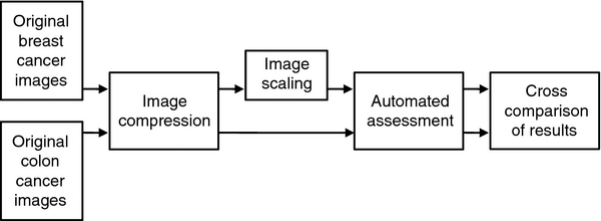
**A flow chart of the image handling protocols used in this study**.

In this paper, the effects of image compression and scaling on automated quantification of IHC stainings, and the effects of image compression on automated tumor segmentation based on texture classification, were studied. An overview of the image handling steps used in this study is in Figure 1. We used publicly available image compression algorithms and a previously described open source image analysis algorithm for quantitative IHC that has been shown to produce results comparable to visual scoring [3]. The other evaluated image analysis method is intended for automated texture-based tumor segmentation using a local binary pattern (LBP) algorithm [13]. The LBP algorithm has been shown to be immune to image compression up to JPG quality levels of 75 in texture classification of natural image series [14].

## Methods

### Patients

#### Breast cancer series for automated IHC analysis

Two hundred patients were selected from a previously reported breast cancer series [15]. The series included tumors from 570 consecutive patients with invasive non-metastatic breast cancer, treated at the Department of Oncology at the Helsinki University Central Hospital between 1997 and 1998. Tumor samples were analyzed using the tissue microarray (TMA) technique. The patients are part of a larger study focusing on hereditary breast cancer, genetic, epidemiological and clinicopathological factors associated with breast cancer risk and prognosis. All patients underwent surgery and were treated according to standard guidelines at that time regarding adjuvant chemotherapy, radiotherapy and endocrine treatment.

#### Colorectal cancer series for automated tumor segmentation

The study is based on 144 randomly selected tissue samples from a series of 643 consecutive patients who underwent surgery for histologically verified colorectal cancer at the Helsinki University Central Hospital in 1989 to 1998 [16].

### Tissue microarray construction

Representative tumor regions in routinely fixed paraffin-embedded samples were defined from H&E-stained sections and marked. Donor tissue blocks were sampled and four cores from breast cancer specimens or three cores from colorectal cancer specimens were punched from each donor block and transferred to the TMA blocks. From the 200 breast cancer tumor samples available, two TMA blocks were prepared, both containing 400 tumor samples [15]. From the 643 colorectal cancer tumor samples available, 27 TMA blocks were prepared, each containing 10-180 tumor samples, and eight TMA blocks were selected for the previous study using texture analysis in identification of tumor epithelium and stroma [16]. Out of the eight TMA blocks, two were randomly selected for the current study. Sections of 3-4 μm were cut from the TMA blocks and transferred to glass slides.

### Immunohistochemistry

#### Breast cancer series for automated IHC analysis

Deparaffinization of the TMA samples was performed using xylene. The slides were rehydrated through graded alcohols to water. IHC for Ki-67 was performed by using Mib-1 antibody (Dako, Stockholm, Sweden) diluted 1:100 in an automated immunostainer (Ventana Medical Systems Inc., Tucson, AZ, USA) using a DAB kit (Ventana). The slides were manually counterstained in Mayer's haematoxylin (Sigma, St Louis, MO, USA). Finally, the slides were dehydrated through alcohol series to xylene and mounted in organic mounting medium (Pertex; Histolab, Gothenburg, Sweden) [15].

#### Colorectal cancer series for automated tumor segmentation

The tissue samples used in the current study were previously immunostained as part of a separate study on the expression of the epidermal growth factor receptor (EGFR). Of note is that this particular immunostaining is not relevant with regard to the objectives of the current study. For IHC of EGFR a Lab Vision Autostainer TM 480 (LabVision, Fremont, CA) was used. Deparaffinised formalin-fixed, paraffin-embedded tissue sections were heated in the pre-treatment module of the autostainer in TRIS-HCl pH 8.5 buffer (for 20 min at 98°C). For inactivation of endogenous peroxidases, the sections were incubated (for 5 min) in Peroxidase Block Solution (DAKO, Carpinteria CA) and incubated for 30 min with the primary antibody NCL-EGFR (Novocastra, Newcastle upon Tyne, UK), diluted 1:10. The sections were then reacted (for 30 min) using the Advance HRP detection system (DAKO, Carpinteria CA). The reaction products were revealed with DAB and finally the sections were counterstained with haematoxylin (for 1 min) [16].

#### Visual scoring of ki-67 percentage in breast cancer TMAs

TMA slides were analyzed by one of the investigators. All scoring was done under the supervision of an experienced breast pathologist. The percentage of Ki-67 positive breast cancer cells was evaluated in one high-power field (40× objective and a field-of-view with a diameter of 450 μm) in each of the four tissue cores on the TMA. Only unequivocal nuclear staining was accepted as a positive reaction for Ki-67. A minimum of 200 cells was counted in each tumor. All statistical analyses were done using both average and maximal values for each patient. When calculating maximal values for Ki-67 in percentage terms, the biopsy core that had the largest number of positively stained cells out of the four was counted and divided by the entire number of cells from that particular biopsy specimen. To obtain the average value in percentage terms, all positive cells from the four biopsy specimens were divided by the entire number of cells from the same specimens [15].

#### Sample digitization

The breast and colorectal cancer TMA slides were digitized with an automated whole-slide scanner (Mirax Scan, Zeiss, Göttingen, Germany), using a 20× (numerical aperture 0.75) objective and a DFW-X710 camera (Sony, Tokyo, Japan). The pixel resolution was 0.26 μm per pixel. The images were initially stored in an uncompressed Bitmap (BMP) format.

#### Image compression and scaling

The scanned images were compressed to a publicly available ISO Standard JPEG2000 wavelet format with the JVScomp software developed at the University of Tampere and freely available at http://jvsmicroscope.uta.fi/?q=jvscomp. The JPEG2000 format is considered as the most efficient way to store large images produced by microscope scanners [17]. The settings for compression were: lossless, and ratios 1:12, 1:25 and 1:50 for lossy compression. Each of the compressed breast cancer tissue images was furthermore scaled down either to 1:1, 1:2, 1:4, 1:8, 1:16, 1:32, 1:64 or 1:128. Due to the scale-variant nature of the LBP algorithm used in the automated tumor segmentation method, scaling series was not applied to the colorectal cancer series images.

#### The virtual microscopy platform

The compressed virtual slides were uploaded to our web server (http://www.webmicroscope.net) running image server software (Image Web Server, Erdas Inc, Atlanta, Georgia). Virtual slides on the website can be viewed and processed with image analysis algorithms (i.e. ImageJ and MATLAB) using a standard web browser interface. The user can navigate into the area of interest in a whole slide sample or TMA, and store the current view as a region-of-interest that subsequently can be processed by image analysis [16]. Each tissue core in Ki-67 TMAs was manually annotated with the Webmicroscope graphical user interface, and exported as losslessly compressed PNG image for subsequent image analysis.

#### Annotation of representative tissue regions for automated tumor segmentation

In the digitized tissue microarray slides, representative areas of each tissue subtype, i.e. stroma (n = 138) and epithelium (n = 269) were defined using the annotation tool described above. The training of the algorithm was carried out as previously [16]. Regions-of-interest are stored in a database and available at http://fimm.webmicroscope.net/oncotexsupplements/epistroma. Image annotation was carried out by one of the researchers (N.L.) and verified by a pathologist (S.N.).

The annotated areas were saved as losslessly compressed PNG images. The dimensions of the annotated areas varied between 168 to 1191 in pixel width and 168 to 1190 pixel height. Magnification was constant i.e. images were always of the same pixel resolution although the image size of the annotations was variable [16].

#### Computer vision algorithms

##### Automated scoring of IHC stainings

The computer vision algorithm used for automated scoring of immunohistochemical stainings in this study is entitled IhcJ [3]. It utilizes the macro language of an image processing and analysis software, ImageJ, which is open source and available for multiple operating systems at http://rsb.info.nih.gov/ij/. The IhcJ algorithm first divides the acquired image of the IHC stained specimen in RGB colour space into separate colour channels by a colour deconvolution method. The ImageJ plugin for colour deconvolution has a built in vector for separating haematoxylin (H) and DAB stainings. After colour deconvolution, H and DAB images are processed separately. By using five random test samples stained for Ki-67, suitable global threshold levels for H and DAB were determined manually. These thresholds were used on both H and DAB images, respectively, and kept constant for the analysis of the main image dataset. Thresholding creates binary masks of H and DAB positive areas and the two areas may overlap. Binary masks were merged into a single result image. In the result image, the area of H-positive and DAB-negative pixels is pseudocoloured with green. The area of DAB-positive pixels regardless of H-status is pseudocoloured with red. The background, where both values are negative, is indicated with white.

The extent of staining is calculated as the total number of DAB-positive pixels divided by the union of the total number of H-positive pixels and the total number of DAB-positive pixels. The staining intensity is calculated from the DAB positive area, as a mean pixel value of original DAB image. The mean intensity value is scaled to range from 0 to 100% to compensate for the effect of different DAB thresholds in subsequent routine use.

##### The automated segmentation of tumor epithelium [16]

###### Texture features

The local binary pattern operator (LBP) compares each pixel in an image to *P *pixels in a circular neighborhood with radius *R *[13]. The intensity value of the central pixel is used to threshold the surrounding pixels forming a binary code. The original LBP was defined in a rectangular 3 × 3 pixel neighborhood (*P *= 8, *R *= 1) for gray-scale images, but the radius of the operator can be extended to include pixel neighborhoods farther from the central pixel (e.g. *P *= 16, *R *= 2).

Invariance to rotation was achieved by using minimized uniform patterns. When uniform patterns are used, all the non-uniform patterns are mapped to one LBP code. This restricts the amount of possible LBP codes to *P *+ 2.

To capture also the contrast information, i.e. the strength of the texture patterns, the LBP was combined with a rotation invariant local variance (VAR). As for the LBP, the VAR is formulated in a circular neighborhood, often with the same radius *R *and sample points *P *as the LBP. Essentially the VAR represents the variance of the gray values of the surrounding pixels i.e., the sample points.

The joint distribution of the above-described operators is used to merge the contrast with the LBP pattern. To determine the joint distribution, the output VAR is quantized to *Q *levels. The quantization is performed by computing VAR for a set of training images and then dividing the distribution of VAR values into *Q *sections, each having an equal number of pixels. This restricts the size of the joint distribution to (*P *+ 2) × *Q *discrete bins. MATLAB implementations for some of the methods presented here are available at http://www.ee.oulu.fi/mvg/page/downloads[16].

###### Preprocessing of images for tumor segmentation

To extract the texture features, the tissue sample images are first scaled, then converted to grayscale and finally possible background area is removed.

In the current study, images were scaled by a constant of 0.5. The grayscale conversion is performed by computing a weighted sum of the R, G and B components of the color image: 0.2989 * R + 0.5870 * G + 0.1140 * B.

Possible background is removed by creating a binary mask in which the foreground tissue pixels are marked by ones and the background pixels by zeros. In bright field microscope images, the background pixels have high luminance values. These bright areas are removed by thresholding the grayscale image. Structures in the resulting binary mask are smoothed morphologically by closing and eroding the binary image. The binary mask is used later to prune areas scarce of tissue i.e., the background [16].

###### Feature extraction for tumor segmentation

The downscaled images are divided into elements and the classification is performed by processing the elements independently. The elements are defined by sliding a square *of 80 × 80 pixel *window through the image. The window is moved row by row from the upper left corner to the lower right by *40 pixels *at a time, thus creating a 50% overlap. If the area of a background binary mask that corresponds to the area of an element contains 50% or more tissue, the particular element is processed, if not, the element is considered as background, and it is not further processed.

For each element, a numerical representation of its texture is computed using two discrete joint distributions: $LBP_{\text{8,1}}^{riu2}+VAR_{8,1}$ and $LBP_{\text{16,2}}^{rius2}+VAR_{16,2}$. The histograms are concatenated to one (8 + 2) × 8 + (16 + 2) × 8 = 224 bins long feature vector. The Euclidean norm of the feature vector is normalized to one [16].

###### Linear classifier for tumor segmentation

A linear support vector machine (SVM) is used to classify the image elements extracted from the input images. A library for large linear classification (LIBLINEAR) was used to implement a linear capacity constant SVM (C-SVM). The optimal value (300) for the parameter C was established by validation [16].

###### The algorithm output

The analyzed images differed in size (pixel dimensions) and therefore contained a varying number of elements that were classified by the SVM. The average SVM score of all elements in an image defined to which class the test image was assigned (stroma or epithelium). The sign of the classification score, or the decision value, indicates on which side of the decision hyperplane a feature vector lays, i.e. it represents the predicted class. The points near the hyperplane in the feature space are more likely incorrect than the ones that are further from it; hence the absolute decision value can be seen as a measure of the certainty of the prediction. Images with an SVM score lower than -1 or higher than 1 where therefore considered as strong candidates for the respective classes, whereas those closer to zero (SVM score between -1 and 1) were considered as weak candidates. The threshold for the classification into the stroma and epithelium catergories was set to zero [16].

#### Statistical analysis

In order to validate the automated methods, the agreements between the visual and automated methods were estimated by percent agreement and kappa-statistics. For comparison between visual and automated IHC quantification, the continuous visual and automated Ki-67 percentages were dichotomized with a seventh decile cut-off, as previously suggested [15]. The results from compressed and scaled images were compared to results from lossless and non-scaled images with percent agreement and kappa-statistics.

## Results

When the breast cancer sample series was dichotomized according to seventh decile cut-off value, the percentage agreement between visual and automated methods for quantification of the Ki-67 staining was 85% and the Kappa value 0.64 (Table 1). For the colorectal cancer series, the percentage agreement between the visual and automated segmentation method was 97% and kappa value 0.93, suggesting very good agreement (Table 2).

**Table 1:** A contingency table for automated and visual assessment of Ki-67 expression

	Automated (lossless compression)			
Visual		**Low**	**High**	**Total**
	**Low**	115	15	**130**
	**High**	13	42	**55**
	**Total**	**128**	**57**	**185**

**Table 2:** A contingency table for automated and visual segmentation of the tumor histology

	Automated (lossless compression)			
**Visual**		**Epithelium**	**Stroma**	**Total**
	**Epithelium**	264	5	**269**
	**Stroma**	7	131	**138**
	**Total**	**271**	**136**	**407**

**Figure 2:**
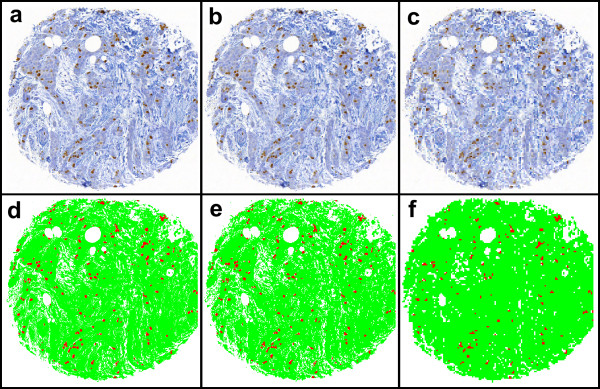
**Sample images of the effect of scaling to visual image quality in a Ki-67 immunostained breast cancer tissue microarray specimen, a) compression ratio 1:50, scaling ratio 1:1, b) compression ratio 1:50, scaling ratio 1:8, and c) compression ratio 1:50, scaling ratio 1:16, with corresponding result images (d-f)**.

**Figure 3:**
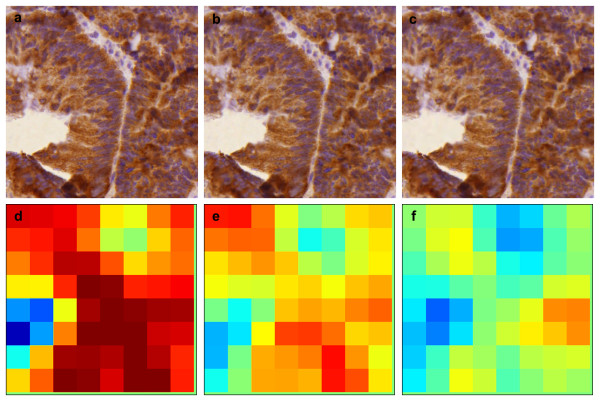
**Sample images of the effect of compression to visual image quality in a colorectal cancer epithelial specimen, a) lossless, b) compression ratio 1:25, and c) compression ratio 1:50, with corresponding result images (d-f)**.

The file sizes of the compressed and scaled images of one of the whole TMA slides from the breast cancer series are given in Table 3. Both the compression and scaling reduces file sizes rapidly. Sample images of the effect of image compression and scaling on image quality are presented in Figures 2 and 3, with corresponding result images.

**Table 3:** File sizes of the whole example breast cancer tissue microarray slide image with different compression (C) and scaling (S) ratios (uncompressed original file size 15540 MB)

[MB]	S1	S2	S4	S8	S16	S32	S64	S128
**Lossless**	4141	1035	259	65	16	4	1	0.3
**C12**	356	89	22	6	1	0.3	0.1	< 0.1
**C25**	166	42	10	3	0.6	0.2	< 0.1	< 0.1
**C50**	81	20	5	1	0.3	0.1	< 0.1	< 0.1

**Figure 4:**
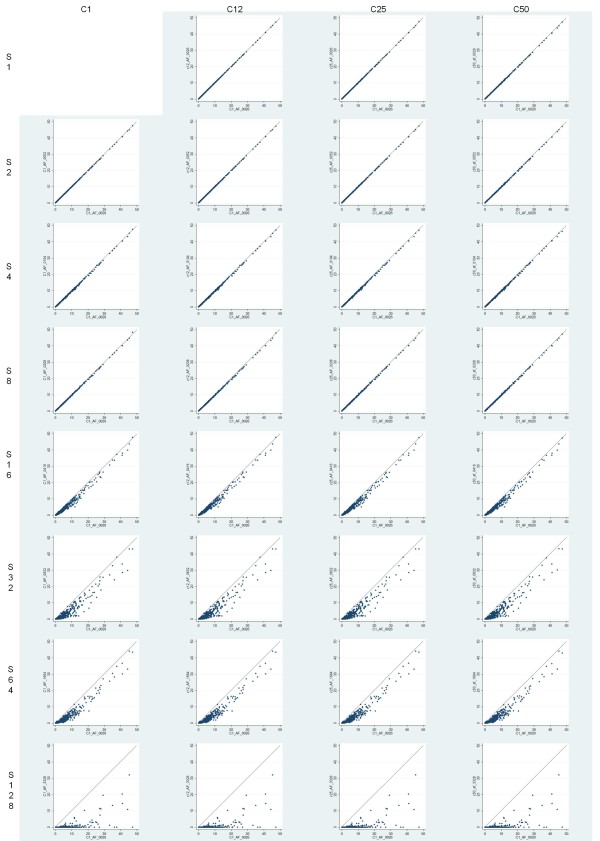
**Scatter plots for different compression and scaling levels in automated Ki-67 immunohistochemistry quantification**.

Considering the continuous results for automated IHC quantification, the scatter plots of each studied compression and scaling levels compared to losslessly compressed and non-scaled results are shown in Figure 4. There is very little difference in results over varying compression levels. When scaling is applied more than 1:8, the results start to deteriorate. Dichotomized results for automated IHC quantification are shown in Tables 4 and 5. The percentage agreements exceed 98% with combined compression ratios up to 1:50 and scaling down to 1:8. Corresponding kappa values stay above 0.96. These results suggest a high level of agreement between aforementioned compression and scaling levels.

**Table 4:** Agreement table for automated Ki-67 IHC quantification (C = compression level, S = scaling level)

[%]	S1	S2	S4	S8	S16	S32	S64	S128
**Lossless**		99.63	98.75	98.87	93.58	86.04	86.51	74.55
**C12**	100.0	99.75	98.75	99.12	93.58	86.04	86.51	74.55
**C25**	99.88	99.63	99.00	99.00	93.71	86.04	86.51	74.55
**C50**	99.13	99.37	98.88	99.25	93.70	86.17	86.90	74.55

**Table 5:** Kappa table for automated Ki-67 IHC quantification (C = compression ratio, S = scaling level)

	S1	S2	S4	S8	S16	S32	S64	S128
**Lossless**		0.9903	0.9674	0.9707	0.8217	0.5715	0.5898	0.0877
**C12**	1.000	0.9936	0.9674	0.9773	0.8217	0.5715	0.5898	0.0877
**C25**	0.9968	0.9903	0.9740	0.9740	0.8255	0.5715	0.5898	0.0877
**C50**	0.9773	0.9838	0.9707	0.9806	0.8254	0.5762	0.6036	0.0877

**Figure 5:**
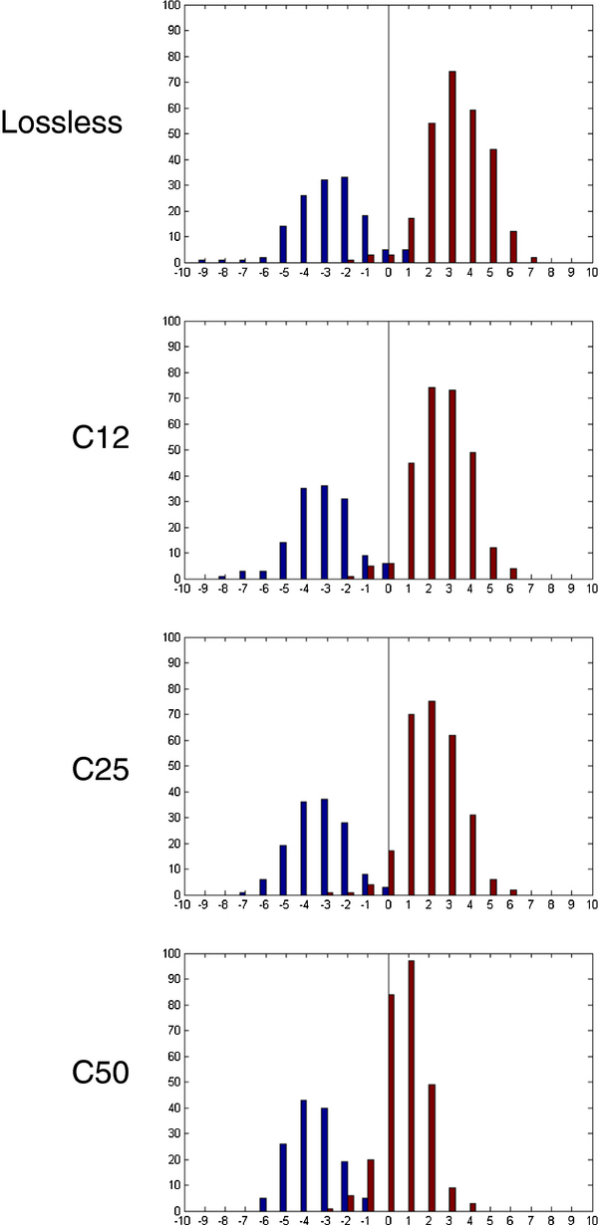
**Histograms of automated tumor segmentation scores (blue denotes stromal and red colorectal cancer epithelial images as determined by visual assessment; negative x-axis values correspond to stroma and positive values to epithelium by decision of automated method)**.

The histogram of automated tumor segmentation scores is shown in Figure 5. The stromal and epithelial images form two distinct peaks in the histogram. When compression level increases, two separate peaks remain but the epithelial peak moves towards the stromal side leading to an increasing number of misclassified images. The effect of this can be observed as a sharp decrease in percentage agreement and kappa values between compression ratios 1:25 and 1:50 (Table 6). By lowering the decision threshold from zero, the discrimination accuracy of the current algorithm can be retained, but would require a re-calibration procedure to be integrated with the algorithm.

**Table 6:** Agreement and kappa table for automated tumor segmentation (C = compression level)

	Agreement [%]	Kappa
**C12**	98.77	0.9726
**C25**	97.05	0.9352
**C50**	85.75	0.7105

## Discussion and conclusions

The results from comparison of visual and automated methods of Ki-67 IHC quantification in breast cancer samples are in line with previously reported results with the utilized algorithm applied to a different data set, which showed a good agreement and also the prognostic value of the results was equal to visual scoring [3]. The storage capacity needed for digital whole-slide scanning of tissue specimens is becoming a major issue especially in pathology institutions with a highly digitized workflow. Image compression or scaling can be used to reduce the need for storage space substantially. The results of this study suggest that images stored for the automated IHC analysis can be compressed and scaled significantly without compromising reliable computer-assisted analysis results. Also the automated tumor segmentation based on local binary pattern texture analysis performed well with images preprocessed with a medium compression ratio. However, these results are likely to be algorithm specific, and may not be applicable to algorithms based on other image features or classification methods. The literature supports this hypothesis, since for example object area measurement does not seem to be affected by image compression, whereas object roundness does [10]. Lossy JPEG2000 compression introduces image degradation in form of slight alterations in color content and blocking [6]. Thus, lossy compression should be performed only once per image in the image processing workflow. If subsequent saving of images is needed, a lossless compression should be used. Scaling may alter the shape of small objects in images, or even lose the smallest objects, due to smaller image resolution. Algorithms that segment small histological entities such as nuclei or algorithms that measure shape of objects may suffer more from image degradations. It has been shown that assessment of segmented IHC stained nuclei in regions of densely packed cells in compressed images is causing significant discrepancy [9]. The IHC algorithm used in this study calculates the percentage of the gross area of stained tissue. Thus, modest image degradation does not seem to affect the algorithm. The LBP algorithm used for tumor epithelium classification is inherently gray-scale invariant, and therefore resists changes in pixel values if the local order of pixels remains unaffected [13].

The automated tumor segmentation method seems to be affected more by the increase of the image compression level than the automated IHC quantification method. For the algorithms used in this study, the authors would recommend using compression ratios up to 1:50 and scaling levels down to 1:8 for automated IHC assessment, and a compression ratio up to 1:25 for automated tumor segmentation. These suggested compression and scaling ratios would reduce the storage space needed for the images to less than 0.03% and to 4.0%, respectively, as compared to losslessly compressed and non-scaled images (Table 3). Since these results might not generalize to other algorithms, the authors suggest each individual method to be validated prior to routine use. Further studies are needed for determination of compression levels sustaining adequate visual appearance, and for different tissue types and other biomarkers. Modest levels of image compression with the JPEG2000 method yields images that seem acceptable in quality by the human observer, whereas higher levels of image scaling lead to loss of details in the images [7]. In conclusion, the storage space needed for digital whole-slide tissue images can be reduced significantly with image compression and scaling, and studied automated image analysis algorithms perform adequately with resulting images.

## Competing interests

The authors declare that they have no competing interests.

## Authors' contributions

JK, ML and JL designed the study and JL was in charge of it. NL identified the regions of interest from the colorectal cancer tissue microarrays and SN confirmed the accuracy. ML wrote the code for the virtual microscopy platform and participated in data acquisition and analysis with JK. JK performed the computational experiments. CH devised the colorectal cancer patient series. CB, HN and KA devised the breast cancer patient series, and KA visually assessed the Ki-67 proliferation indexes. JK, JL and NL drafted the manuscript, and ML, CJ, CB, HN, KA and SN contributed to improving the draft of the manuscript. All authors read and approved the final manuscript.
